# Commitments, Conditions and Corruption: An Interpretative Phenomenological Analysis of Physician Recruitment and Retention Experiences in Indonesia

**DOI:** 10.3390/ijerph19095518

**Published:** 2022-05-02

**Authors:** Farah C. Noya, Sandra E. Carr, Sandra C. Thompson

**Affiliations:** 1 Division of Health Professions Education, School of Allied Health, University of Western Australia, Perth, WA 6009, Australia; sandra.carr@uwa.edu.au; 2 Medical Education Unit, Faculty of Medicine, Pattimura University, Ambon 97233, Indonesia; 3 Western Australian Centre for Rural Health, The University of Western Australia, Geraldton, WA 6530, Australia; sandra.thompson@uwa.edu.au

**Keywords:** medical workforce shortage, phenomenology, rural and remote, recruitment and retention, unethical governance, work motivation and satisfaction

## Abstract

Complex factors influence physicians’ decisions to remain in rural and remote (RR) practice. Indonesia, particularly, has various degrees of poor governance contributing to physicians’ decisions to stay or leave RR practice. However, there is a paucity of literature exploring the phenomenon from the perspective of Indonesian RR physicians. This study explores physicians’ lived experiences working and living in Indonesian RR areas and the motivations that underpin their decisions to remain in the RR settings. An interpretative phenomenological analysis was utilised to explore the experiences of 26 consenting voluntary participants currently working in the RR areas of Maluku Province. A focus group discussion was undertaken with post-interns (*n* = 7), and semi-structured interviews were undertaken with junior (*n* = 9) and senior physicians (*n* = 10) working in district hospitals and RR health centres. Corruption was identified as an overarching theme that was referred to in all of the derived themes. Corruption adversely affected physicians’ lives, work and careers and influenced their motivation to remain working in Indonesia’s RR districts. Addressing the RR workforce shortage requires political action to reduce corruptive practice in the districts’ governance. Establishing a partnership with regional medical schools could assist in implementing evidence-based strategies to improve workforce recruitment, development, and retention of the RR medical workforce.

## 1. Introduction

Residents of Indonesia’s rural and remote (RR) provinces experience many challenges accessing services such as healthcare. In 2020, the Indonesian National Health Profile identified an acute and significant problem with vacancy rates of physicians in RR *Puskesmas* (Public Health Centers/PHCs at the sub-district level) [1]. *Puskesmas* were designed to provide access to primary healthcare services as a first-level health facility [2]. However, the 2020 report identified that 14.1% of *Puskesmas* had insufficient physicians and that the ratio of physicians per population in the underdeveloped regions is 1:67,916 compared to 1:1510 nationally [1], representing an inequitable ratio of 1:45. Most healthcare professionals such as physicians and nurses are trained outside of the RR settings in schools established in urban locations in the western provinces of Indonesia [1,3], although a rural background and rural-based training are two of the strongest factors influencing the intention of physicians to practise rurally [3,4,5]. The challenges of geographic spread, low population density, limited infrastructure, as well as the higher costs of delivering RR health care [6,7] affect important health outcomes, especially maternal and infant health, with low achievement on indicators [8] for the World Health Organisation (WHO) Sustainable Development Goals (SDGs) targets [9,10]. These factors and RR health workforce retention are global health challenges documented by the WHO [11].

Indonesia’s rural and remote areas experience difficulties with development and utilisation of healthcare facilities [12,13,14]. Variations in local governance due to decentralisation allow different levels of development and are influenced by politics and clientelism [15]. In Maluku Province, the health status key indicators, challenges with abandoned development projects and limited infrastructure for education, health and transport, plus district autonomy in health planning, are similar to conditions reported elsewhere in Indonesia [1,12,16]. One strategy to improve the situation, applied since the 1960s, has been the provision of temporary health workers, with regular changes in terms and conditions, from compulsory service to the current voluntary Nusantara Sehat (a temporary health employment scheme funded by the Ministry of Health) [17,18,19,20]. Both the Indonesian National Health Policy regarding temporary employment (Pegawai Tidak Tetap/PTT) and Nusantara Sehat (NS) emphasise continuing availability of service in all divisions of healthcare workers in remote and underserved areas [1,17]. However, a chronic shortage of the medical workforce remains [1,17], suggesting that challenges still exist for those working and living in RR areas of Indonesia despite the strategies applied.

Living and working in RR areas presents challenges for physicians, not just in their professional practice but also in their own and their family’s personal living experiences [21,22,23]. These physicians struggle to provide safe and quality health care in the environment in which they work. They also face the personal impacts of isolation and remoteness in their living conditions. The challenges of living and working remotely are recognised as contributing to the long-term unsolved problems of recruiting and retaining RR physicians in Indonesia [3,4]. A survey of physicians in RR practice in Maluku found many were in temporary positions and were less likely to stay in RR practice [3]. Other factors that influence physicians’ decisions to remain in practice in the RR areas appear to be complex. For example, a case study of four districts in Indonesia revealed various degrees of poor governance through a lack of transparency and low accountability, provision of unsafe health services, and profit-oriented management as the contributing factors influencing physicians’ decisions to stay or leave [24]. However, there is still a gap in the literature exploring the phenomenon of attraction and retention of the RR medical workforce from the perspective of Indonesian RR physicians. Exploring the stories of the medical workforce in RR Maluku may offer insight into the challenges and solutions needed to improve the RR workforce and, thereby, the community health status. 

This study explored the lived experiences of physicians living and working in the RR Maluku Province to understand their decision to remain in rural practice. The research aims to inform key district and national stakeholders about the barriers and facilitators to working in RR communities and political and practical actions to improve the chronic problem of physician shortages in rural and remote areas.

## 2. Materials and Methods

### 2.1. Study Design

This study used an interpretative phenomenological analysis (IPA) [25] to explore the lived experience of physicians who work in the RR areas of Maluku Province and why physicians choose (or not) to remain working in the RR Maluku Province. The researchers brought their unconscious thoughts to the surface rather than allowing them to unconsciously influence the research. IPA allows for the participants’ experiences to be central and the researchers’ experiences also to be acknowledged [26]. 

### 2.2. Setting and Sampling

The study population were medical graduates who currently work in Maluku Province. Purposive homogenous sampling was used to identify participants for whom the research question was significant to achieve three participant groups. Thirty-six physicians from different districts across Maluku Province with differing lengths of RR service were invited:

(1) Post-intern physicians who chose not to continue working in the RR area. They were recruited from a population of medical interns who worked in the Maluku province during 2019. During their one year of internship for mastery and independent practice, they were placed in a district hospital (8 months) and a *Puskesmas* (4 months).
(2) Junior physicians (≤5 years of working experience) who were Pattimura University alumni who continued working in the RR workforce. They were recruited from a population of junior physicians working in Maluku RR areas at district hospitals or *Puskesmas* in sub-districts.
(3) Senior physicians (>5 years of working experience), including medical specialists who are currently working in the RR areas in Maluku Province, were recruited from a population of physicians who were practising in RR Maluku Province. Some worked at district hospitals, others at *Puskesmas* in sub-districts.

### 2.3. Data Collection

The interviewer (F.N.) engaged respondents from December 2019 to February 2020 in Ambon, the capital of Maluku Province, Indonesia, with individual semi-structured interviews undertaken with junior and senior physicians. A separate focus group discussion (FGD) was undertaken with post-intern physicians as there was limited opportunity to speak with them individually. Although controversial for data collection in phenomenological studies, the focus group can be advantageous in IPA. For IPA study designs, the focus group can offer richer data from participant interaction, constant clarification of perspectives, and validation of key points between the researcher and the participants [27].

Discussion and interviews were completed in Indonesian, audio-recorded, and transcribed verbatim. The transcriptions were later translated to English by professionals to allow analysis and interpretation by the other researchers (S.C. and S.T.). 

### 2.4. Interview Guide

Semi-structured interview questions were utilised to collect data as they allow dialogue whereby initial questions can be modified to expand on participants’ responses and explore interesting and important areas that arise [25]. The list of semi-structured questions was developed based on the research questions and examples from the literature [25]. The list of questions guided the discussion about the participants’ experiences and intentions and included questions about the participant’s current work location, their lived experience of being in the RR medical workforce, and their motivation to work rurally (Table 1).

### 2.5. Data Analysis

This IPA utilised the seven steps of the data analysis guide introduced by Smith et al. [25]. The process includes reading, re-reading, noting, finding patterns across the data, and interpreting the data. This approach includes analysing the data for meaningful phrases, developing meaning and collating them into themes, and presenting an exhaustive description of the phenomenon [25,28]. 

F.N. started the data analysis by listening to the interview recording while reading the transcript. Multiple readings of individual transcripts in the Indonesian language were done by F.N. and later in English by F.N., S.C. and S.T. The research questions and objectives informed and guided the coding process. Annotation and the following steps were undertaken in English. 

Themes from all transcriptions were juxtaposed and clustered together across the cases. The subordinate themes were collated, and extracts gathered from across the interviews. Two researchers (F.N., S.C.) worked to define the meaning of the subordinate and superordinate themes. The third researcher (S.T.) clarified and confirmed identified subordinate and superordinate themes with the research team. All researchers then agreed on the final superordinate and subordinate themes. The inductive and iterative process helped the researchers develop perspectives and ensured that these perspectives from the RR physicians and their interpretation were contextual and relevant [29]. The authors brought some of their own experience to the interpretation of data and were mindful of setting aside their personal biases to ensure interpretations were consistently demonstrated in the participants’ perspectives to ensure credibility.

### 2.6. Trustworthiness and Rigour

The lead researcher and interviewer (F.N.) is a physician and medical educator with experience in living and working in remote areas of Indonesia. As the lead analyst, she is well placed to make inferences from data to persons [29] and to ensure fidelity in the fit between respondents’ views and the researcher’s representation of them [30]. The interpretation utilised reflexivity [30], and inferences were made with caution and awareness of the Indonesian contextual and cultural basis on which the data is generated, while recognising that meaning, cognition, influence and action are open to interpretation. 

Analysis was developed based on the transcripts, with extracts used to ensure the voice of the participants was represented. The inductive and iterative process helped the researcher to establish the “insider perspective” (phenomenologist) and to offer an “outsider interpretation” (interpretive) [29]. This analysis prioritises the participants’ viewpoints at the core of their story and surfaces their experiences and concerns to explain their workforce choices from their RR workforce exposure [29]. 

To ensure the trustworthiness of the data, the authors established careful, systematic immersion in the data through multiple readings, listening to the recording, and detailed coding [30]. The authors met to debrief to check preliminary findings and interpretations against the raw data to ensure that the research process was logical, traceable and documented. The rigour of the study was maintained by the appropriateness of the sample to explore the research question, as did the use of semi-structured questions and the completeness of the analysis undertaken [25]. The study sample was selected carefully to match the research questions. The interviews utilised a semi-structured guide with questions and probing that consistently focused on important cues to explore the underlying meaning. The analysis was interpretive to elicit the important emerging themes. The overall project was cross-checked against the IPA quality evaluation guide developed by Smith, 2011 [31]. 

### 2.7. Ethics 

Ethical principles were adhered to with informed consent obtained from participants before the interviews. Alphanumerical codes were used where quotes were abstracted to maintain participants’ confidentiality. Ethics approval to undertake this study was granted by the University of Western Australia Human Research Ethics Committee (UWA HREC) and the Pattimura University HREC. 

## 3. Results

### 3.1. Overview of the Respondents

Of the 36 physicians invited, 26 consented to participate, representing nine of the 11 districts in the Maluku Province. The physicians had been working in Maluku for varying lengths of time. Seniority varied from post interns (PI), junior physicians (JD, ≤5 years working experience), and senior physicians (SD, >5 years working experience), including specialists (S) (Table 2). Most physicians were female (65%) with permanent employment (65%). Each participant was allocated an alphanumeric code to ensure that the individual’s identity was protected and to represent their employment status and level of seniority.

**Figure 1:**
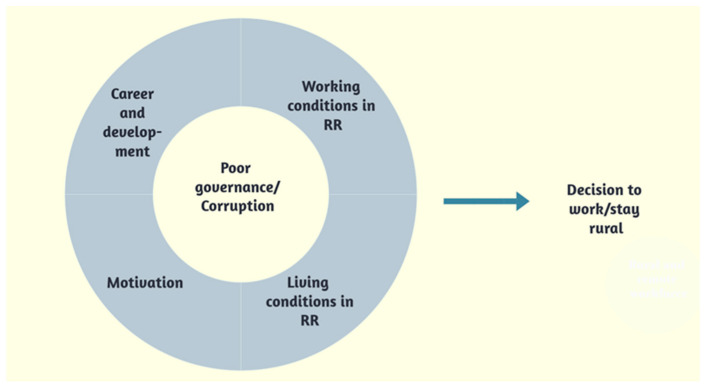
Thematic map.

Five superordinate themes emerged as the interpretation of the descriptive experiences of the participants: poor governance, working conditions in RR areas, career and professional development, living conditions in RR areas and motivations to work in RR areas. Corruption was identified as an overarching theme as it was referred to in all of the derived themes (Figure 1). Facilitators and barriers to working in RR were identified across the emergent themes and contributed to participants’ decision-making with respect to working rurally (Figure 1).

### 3.2. Theme 1. Poor Governance 

Participants were overwhelmingly scathing about aspects of the governance of the RR health system and provided detailed descriptions of poor governance at every level: District government, District Health Office (DHO), district hospitals or *Puskesmas*. With few exceptions, the implications of poor governance issues were raised in the focus group of post-internship physicians and continued to resonate through the individual interviews with more senior general practitioners (GPs) and specialists. 

#### 3.2.1. Favouritism

Predominantly, participants talked about relationship problems in leadership at various levels. Leaders of DHO and *Puskesmas* were reportedly appointed based on political affinity with the district’s highest leadership and without consideration of their leadership abilities and supporting qualifications. A junior physician described this situation in their district:

*In general, the head of the Puskesmas graduated from SPK (Sekolah Perawat Kesehatan: Nursing School, high school level). The leader of the Puskesmas in this district has always been politically oriented.* (JD2)

Participants provided examples of how the DHO and *Puskesmas* leaders’ political appointments underpinned the misunderstanding of responsibilities and led to poor management:

*…people with theunclear capacity to become the head of the Puskesmas were kept there… I don’t know, maybe to let all the rotten things happen.* (PI1)

The junior physicians raised favouritism as influencing the allocation of placement locations:

*Usually, physicians whose families have direct access to the leadership there were assigned in the city. In the center of the district, on the main island. But, if you don’t have anyone there, you will be automatically placed far away…*(PI4)

Post-interns also identified favouritism occurring during their internship.

*If there is a high-rank official’s child in the internship group, everything will run smoothly, and incentivescan also be suddenly provided or increased.* (PI3)

Support being stopped if one offends the leadership was another example of unfair treatment:

*Because the previous batch of interns caused problems, our batch had to bear the bitterness. The official house, car, and all needs were immediately cut down. And the incentives were cut by more than half.* (PI4)

The closeness of the *Puskesmas* leader to the DHO and the Regent could also mean the *Puskesmas* received more support, for example, in the procurement of medical equipment and facilities compared to other *Puskesmas*. If a community member had a close relationship with high-ranking officers in the regency, participants reported their ease in making complaints about physicians and medical services or obtaining support.

*If elected council members from the area, they can voice their aspirations to the top. If there is no representative, it’s a bit difficult to move forward. Now, because there is a council member from there, Puskesmas … can be facilitated.* (JD4)

#### 3.2.2. Poor Supply Chain and Human Resource Management

A lack of commitment from the DHO and the district government was identified as a barrier to quality service. Lack of medicine and equipment created a considerable strain on physicians trying to deliver adequate patient care. Medical supplies orders were placed by *Puskesmas* or the hospital, but not followed up, and supply was apparently neglected.

*For patients with cases of angina pectoris, we also experienced the limitation of ISDN (Isosorbide Dinitrate, Vasodilator for Angina). It is essential medicine yet lacks in stock. Even though they had reported it to the Department, it turned out that they were not followed up.* (PI3)

Poor conduct was also identified in human resources management. The participants mentioned that the DHO relied on temporary solutions, recruiting physicians and specialists with temporary contracts. Apart from district-funded temporary placement for physicians, the district government kept on applying for a central-government-funded program for temporary contract physicians or visiting residents from universities where physicians only are onsite for three months to two years. Some post-interns reported they were keen to work in RR areas but that their application for work in the district had been neglected.

*We are very interested in serving this area. However, when we finished the internship, it turned out that they had just accepted temporary physicians. The lure is that they will select the second batch in about 1–2 months. So, we applied and waited for the call. There was no call at all after more than six months. So we tried to go to another area.* (PI5)

Such poor recruitment approaches undermined participants’ ability to work in a remote setting; as another post-intern described, there was a lack of transparency in district-level workforce planning.

*I have encountered such problems and have tried to report it according to the procedure, but there was no follow-up again. Hence, it doesn’t mean that we don’t want to go to remote areas, but it was attributed to the fact that there was no follow-ups. It turns out that they (DHO) actually propose the district’s remote areas to be filled by Nusantara Sehat (NS) Program.* (PI3)

Turnover in specialist advice because of temporary appointments created its own challenges, as one post intern described:

*We needed to re-adjust to different treatment and consulting styles.* (PI5)

#### 3.2.3. Complicated Bureaucracy 

Bureaucracy was pointed out as overly complicated for the procurement of essential medical equipment in the district hospital, with extended processes and timelines to obtain equipment.

*The point for physicians is the equipment is needed and important for the service. But, it turns out thatthe flow is complicated because it has to go through the local government, then it was discussed again in the DPRD (Local council) before can go for tender.* (SD6)

Late payment of financial incentives was another challenge that affected the physicians’ living standards without adequate accountability or care for the impact on physicians. This severely affected the physicians’ ability to support themselves with basics:

*Fortunately, here you can still ask parents for help. If there are no parents, who would you ask for help dealing with it? Yes, you have to be smart to cut other expenses and prioritise food. Because if you don’t eat, you won’t be able to take care of the patient.* (JD8)

A junior physician with a temporary contract had to wait for eight months before receiving their salary:

*Maybe that is also one of the factors that made me stressed there. It was hard to live there. Far from the city, gasoline per litre is IDR. 20,000. Imagine every day I went back and forth using my own expense because accommodation was not provided. I did not receive the salary and incentive starting April to November. I never got any funds. I kind of want to cry. I called the DHO, but no response back. I said, “Oh, this is enough!” I think, if until December I don’t get my salary, I will stop. Useless! Our service is manipulated, but no appreciation. It’s really a service. Then, in December, I received it all at once. What can I do? Just be grateful.* (JD6)

Even specialists reported concerns with late payments of the incentives.

*Actually, as long as it’s not delayed, our incentives can be sufficient. But, if you have to wait up to a year, it meansyou have to borrow first for your needs. We aren’t rich people. Once the incentives were received, a big portion was used to cover debts. Moreover, I am also pending private practice and practice in another hospital during the pandemic. So income is reduced, plus late payment. It’s hard for us*. (S1)

#### 3.2.4. Fraud and Bogus Offers

Many participants identified problems with corporate governance processes, including data manipulation or fraudulent reporting:

*The reports were fictitious. Data were manipulated in such way. Puskesmas staff and DHO hada collusion in the verification of the report. Proof of purchases made available. So, it’s all manipulated.* (PI1)

The procurement of health facilities also caused major concerns with a very apparent example of the problem described:

*Before the accreditation of Puskesmas, DHO assisted all Puskesmas in the form of a wastewater treatment plant (WWTP), from septic tanks to biotech. But not including the installation. The septic tank was left on the ground. The important thing for them was that the contract had been approved,and the WWTP had been sent to the Puskesmas. Until recently, our infectious wastes are still mixed with general wastes and burned in previous usual places.* (JD1)

One junior physician reported feeling cheated by the difference in the advertised salaries to encourage physicians to come to the rural district and what was actually paid:

*Thegovernmentthere seemed to sell [the RR opportunities]. It can be quite a big incentive (IDR 30,000,000). In fact, it wasn’t what we got. The incentive was IDR. 6,000,000. What can I say?* (JD5)

#### 3.2.5. Work Orientation of Colleagues and Management

Participants often identified that management was more oriented towards meeting contract requirements than community health needs. For example:

*They only care about getting down to doing activities because BOK (Bantuan Operasional Khusus: Special Operational Assistance Fund) and JKN (Jaminan Kesehatan Nasional: National Health Insurance) funded it. Will these activities have any impact on the community? They don’t think about it. The funds were the concern. So, the negativeimpressionfor me is that they were money-oriented.* (JD1)

### 3.3. Theme 2. Working Conditions in RR Areas

#### 3.3.1. Physical Working Environment

The poor physical working environment was raised regardless of participants’ duration of experience and workplace settings. Recurrent issues identified were inadequate facilities, equipment and supplies for emergencies inpatient and outpatient services, across both the *Puskesmas* and hospitals. Some districts described had *Puskesmas* without electricity and a clean water supply. Even though a generator was provided in the *Puskesmas*, the fuel was not always supplied. As a junior physician noted regarding the building and water supply for the *Puskesmas*:

*The building is very old. Looks like it has weathered. Also, there is no water; this is a pity and wrong for Infection Prevention and Control. There is no sink and clean water, only like handsanitiser.* (JD8)

The equipment and facilities for medical treatment were of concern for most of those interviewed. This problem occurred even in district hospitals to which referrals from *Puskesmas* were directed.

*There is no emergency handling procedure because the facilities and equipment at the hospital are not much different from the Puskesmas facilities. This [despite that the] district hospital was just yesterday under accreditation.* (JD1)

Shortages of equipment and medicines were regular frustrations. One post-intern commented on the adequacy of emergency supply at the hospital:

*…the easiest example is 40% glucose. When there is a hypoglycemic patient, looking for it is like looking for gold in this hospital. Very difficult. In fact, I can say, “Yes, just sugar.” I think it is easier to find a lost gold coin than to look for G40 at (….) Hospital for hypoglycemic patients. Most often, there are only two in the neonates’ room, and if borrowed, it must be returned quickly.* (PI5)

A few participants were satisfied with the existing facilities as long as medical procedures and treatment could be delivered. Nevertheless, they hoped for improvement for better service coverage.

*In all the limitations of equipment, at least we can do emergency operations. But minimally invasive advancedprocedure is difficult to carry out with our limitations.* (S1)

Another senior specialist was pleased that all the required facilities and equipment for their specialist service in the hospital were available. The management was committed to ensuring that all services related to existing specialist areas in the hospital occurred.

*When I cameback from my specialist training, I found that most facilities and equipment have been provided. Some others I requested later were also granted.* (S2)

Participants raised another work challenge related to problematic patient referrals, with referral processes pertaining to the islands’ available transport and weather conditions making decisions more complex. As a junior physician commented:

*…If weforce referrals, the patient could die on the way. Besides bad land transportation, we also have to cross the sea; so, we must consider whether it is possible to travel by speedboat and other related conditions.* (JD3)

Many physicians expressed considerable concern regarding the lack of comfort and security in the work environment while working. This concern reflected genuine anxiety related to their safety. A senior physician captured this discomfort and concerns regarding security when caring for patients in criminal cases:

*…in the ER (emergency room) when there is acase of someone getting stabbed. If there is a fight, they (anyone) can enter (the ER). For the protection of the physician, there is a security guard and an army. But, at most, they can only reprimand because if they do something tougher, it would be a problem. So, it is hazardous for us, for physicians and nurses. And it has happened again and again.* (SD3)

#### 3.3.2. The Community’s Health Awareness

Low health literacy in the community meant health behaviours lead to low health-seeking consultation and awareness of health, which could be challenging for participants:

*There wasa TBC (Tuberculosis) patient in an indigenous community who had a bad cough. But he didn’t want to go to the Puskesmas, let alone give a sputum sample or check-up. They feel disgraceful if other people know their plight.* (JD1)

On the other hand, participants described satisfaction when they experienced changes in community health awareness upon physicians working in the area:

*When I first came, only 1–2 patients visited the Puskesmas.After many people found out [about the physicians], visits to Puskesmas began to increase. Emergency patients were immediately taken to the Puskesmas. No more delaying. Even though there was still some resistance.* (JD4)

#### 3.3.3. Incentives from the Workplace

For some physicians, significant financial incentives were an advantage of RR work, influencing their decision to stay. However, the participants reported significant inconsistencies with the incentives for similar workloads in one district due to different recruitment schemes. Participants who worked under the Ministry of Health (MoH)’s temporary scheme and interns who were paid by MOH received more significant financial incentives. Besides the sum from the MoH, they were also paid by the districts. In contrast, participants with local temporary contracts received smaller financial incentives. This junior physician highlighted the inconsistency of such arrangements:

*…there is also an NS at this Puskesmas. The NS has a high salary, even though the work is the same. Sometimes, when I think about it, uh, it’s better to wait for NS. But, then it willbe like…thinking about money only.* (JD6)

### 3.4. Theme 3. Career and Professional Development

#### 3.4.1. Quality of Training

In relation to the training for interns, participants identified inadequate facilities and equipment as barriers to the quality of their training and the struggles they experienced with providing suboptimal care when they knew better care was possible.

*Medical treatment and management of the Puskesmas in the city are in line with what we learned duringour education. But, the knowledge doesn’t fit in the district. We want to implement what we learned first, but if the facilities and other things don’t match, it feels so wrong.* (PI3)

The post-interns identified limited supervision due to a shortage of supervisors, reflecting a lack of clinical governance:

*Many cases require us to rack our brains. There are no standard procedures. The clinical pathway is up to the person in charge. It’s up to the specialist or the general practitioner who treats the patient in the ER. There is no consultation reference.* (PI5)

#### 3.4.2. Professional Support

Regarding support during their service, junior physicians commented that they sourced their own support from more senior or specialist physicians at more advanced facilities. However, these efforts often relied upon connecting to these experts at critical times. There was an intersection with poor infrastructure as they were sometimes hindered by limited or unreliable network coverage.

*In emergency conditions that cannot be handled, I call a specialist I know,…in one case, every treatment had been administered, then when I want a consultation, the network signal is troubled, I have to go to the ferry dock, approximately 150 m from the hospital. When you place a call, you can’t move. Otherwise, the network is disconnected.* (JD5)

#### 3.4.3. Opportunity

The lack of opportunity and support were identified as a barrier to professional development:

*Only certain people were given the opportunity for postgraduate training. I once took the test butwasn’t recommended by the Regent. The recommendation from the regional head is a big point. So, there are many disappointments, but I just live it.* (SD6)

Another senior physician identified that workforce shortages negatively affected their opportunity for professional development. The staff scarcity hindered them from leaving the area to take specialist training admission tests:

*There are few physicians here, so it’s a bit difficult for us because specialist tests take at least a month. When we were in the process of the test, the management called us to come back. It breaks concentration. Not comfortable. In the end, never mind… [Test discontinued].* (SD3)

However, a few participants reported they had found opportunities and support from their district, even receiving a recommendation for specialist training with a scholarship to support the tuition:

*I foundthe opportunities were open to all for scholarships and postgraduate training recommendations. But we have to secure admittance to the targeted program by ourselves. I am blessed to have taken only one shot in the exam to be accepted*. (S1)

#### 3.4.4. Career Pathways in RR Areas

Regarding career pathways, different preferences were expressed. For some participants, their preferred career plans were not compatible with the RR conditions, post-intern intent on academic and research pathways. Another junior physician brought up their mismatch with the poor management situation of *Puskesmas* in RR areas and felt it would be appropriate to join an organisation with effective management so that their work would have more impact on the community.

A junior physician who intended to stay rurally expressed concern about having to leave the post to pursue specialist training. This was because the RR position would not be filled, and *Puskesmas* was not compatible with specialist services:

*That is the dilemma. Either keep serving there and sacrifice my capacity building or the other wayaround.* (JD3)

This concern was reiterated by a specialist who felt that even though their specialty was needed in the district, some services could not be provided at the district hospital despite their qualification:

*The equipment is not provided until now...my skills are like waste, not optimal. I stayed because of the specialist services needed to improve patient care.* (S2)

### 3.5. Theme 4. Living Conditions in RR Areas

#### 3.5.1. Physical Living Conditions

The lack of basic infrastructure and the unsatisfactory conditions for living and working in these regions was expressed by most participants who felt their basic needs were not met. Accommodation was regarded as a basic need that the DHO should be responsible for, but in fact, may not provide. When it was provided, the quality of the buildings, furnishings, size and need for sharing were reasons for complaint. Common issues related to the age and condition of the housing and the lack of basic infrastructure. Their management did not address longstanding problems:

*I’ve been living in a rented house at my own expense. No official residence was provided. The official house is an old building and has been damaged because it has never been occupied. It wasnever repaired, although there was a request since 2–3 years ago.* (SD2)

The RR physicians reported poor living conditions with a lack of basic needs such as water, food and electricity:

*The electricitythere was on for only 12 hours. No electricity during the day.* (JD9)

There were reports of inadequate telecommunication and internet access, no food market and high food prices. A participant noted the lack of a clean water supply:

*We ask people to carry waterto the house because the water source is far away and still uses a spring from the Dutch era. The spring is behind people’s houses, and the pipes are small. That’s (water supply) for a village. The government has built pipelines, but they were always damaged. So, yes, we buy water every day.* (JD8)

Another challenge was geographical remoteness and the poor quality of roads and transportation:

*The road is much damaged. The puddles can reach knee-deep if it rains. It’s also difficult to use a vehicle because of theslipperyroad and steep ramp. The path is arduous.* (JD5)

Distant islands and servicing outer regions can require travelling over a considerable distance with only substandard transport available and inadequate attention to safety:

*The sea access was really challenging. We would have to go around the island. That’s far away. However, the local government has no concern for the safety of transportation. Most of thespeedboats operated were privately owned, and no one monitored and reminded them of safety concerns when the waves were high.* (JD3)

#### 3.5.2. Community and Safety

The poor attitude and behaviour of community members was a complaint of participants. Drinking of alcohol was identified as a problem. One junior physician described sexual harassment experienced by a work colleague from a drunken villager and raised multiple issues related to safety:

*To make a call, we have to go finding for a signal, near a ravine, near durian trees where there is no house at all. It’s very dark at night. We use a time limit to make a call—people in thisvillage are likely to get drunk, so we have to be careful. Once, my female midwife, riding a motorcycle, some people were drunk. It was still 12 km away (from home). A man showed his penis to her. Reporting to the police is not worth it; at most (the perpetrator) is just (asked to) make a statement. We fear that something even worse will happen.* (JD8)

However, some participants described the welcome and appreciation they received from communities, and that they have learned valuable life skills living in RR areas, sometimes driven by necessity:

*Even though we have money, it is difficult to find an eatery. This taught us something unique; to be independent in meeting our daily needs, for example, cooking ourselves and growingour vegetables because there is no market.* (SD5)

### 3.6. Theme 5. Motivation to Work and Stay in RR Posts

#### 3.6.1. Altruism

A powerful sense of responsibility and obligation as Maluku descendants to improve community health status was reported by participants for serving in Maluku RR areas, including post-interns who had left the RR postings:

*Physiciansshouldbe ready to serve wherever places need us. Although I wasn’t born here, I am a descendant of this district. Sometimes it is not all about the money but the joy of seeing my people happy.* (S1)

Professional expectations of the improved health status of the district, the joy of serving and seeing people healed and happy were other reasons mentioned. The sense of being able to contribute to change and health improvements was a key driver of serving there:

*Some things consistently wrongly implemented can actually be changed… There was pride in overcoming problems with limited facilities and medicines. Also, gratitude from patients when they can recover. In essence, I want to serve and give the best to the community withwhat is in me.* (SD5)

A sense of belonging to the area helped some participants endure and remain in the RR setting:

*I am a local woman, and thus I feel more comfortable working in my own area. I am already used to its customs, habits, and language. Actually, those factors make me feel at home. IbelieveI have never experienced any negative experiences. It is just related to technical problems, which are commonly encountered in work. My parents are here, my family is here, and I am also married to the local person of this area. So, those are some factors that make me feel at home.* (SD4)

Despite having no attachment to a particular district through family or bloodline, some participants chose RR postings as an expression of gratitude to God for their blessed life,

*…it is my thanksgiving to God. He has given me the immeasurable, also the opportunity to becomea physician.* (S2)

#### 3.6.2. Attractors and Detractors from the RR Areas

Factors that acted to attract or dissuade participants from ongoing work in RR areas were integrated into all other superordinate themes. Other than altruistic intentions to serve, some participants were attracted by the financial incentives offered:

*The more remotethe area, the greater the incentive. That’s a promise from the district. I’m definitely interested in it. Everyone is interested. For example, NusantaraSehat (a temporary health employment scheme funded by the MoH)from the Ministry was given incentives from the district of IDR 3,600,000-IDR. 3,800,000 per month apart from their salaries of tens of millions. With that sum of money, we must be very tempted.* (PI5)

For some participants, appreciation from the community and colleagues brought comfort and satisfaction:

*We were actually more respected in rural areas. To be honest, in Ambon, we are not as appreciatedas when we were there. What we conveyed became something precious for the community and other health workers.* (PI5)

However, there were detractions to remaining in practice in the RR. Concern about personal safety meant some physicians had reconsidered continuing RR work:

*I personally experienced one incident related to racial elements directly from the patient’s family in front of me and some nurses. So, I thought again. If one person is like this, what is his representation in society like? I was rethinking about going back there again.* (PI7)

Some participants felt unappreciated by the community regardless of their good intentions and service. As stated by a senior doctor:

*Because I care, I want to check a patient I suspected to have HIV. But, my house suddenly got stoned. Because (HIV-AIDS) is a big scourge in society, those who are just suspected of having HIV will be ostracised. Their extended family didn’t accept it. They even wanted to report me to the police. I never meant to trouble them. I just want to help—why is it so difficult?”* (SD1)

For some participants who had previously shown enthusiasm to serve in RR settings, unethical governance undermined their altruism and idealism and made them rethink remaining in RR practice.

*I still want to serve in the areas, apart from deviations and unfairness in the health sector in Maluku … due to some factors in politics, facilities, and assurance. Even though we have served there for years,there is no guarantee to become a specialist … To be honest, doctors in Ambon are not as appreciated as those serving in the regions … However, everyone has their own way of life and destiny. Some are stuck, some are still, hopeful, some are still waiting for an answer. Personally …[I] still care for serving in remote areas … I believe that most of doctors want to serve in the remote area. But there are too many deviations from reasonable standards, such as political, financial, and conflicts of interest.* (PI1)

### 3.7. Summary of Findings

The authors summarised the participants’ lived experiences into the key concepts of “commitment, conditions, and corruption.” “Commitment” points to the broken government commitment to fulfil the rights and expectations of physicians working and living in remote areas and to the altruism and professionalism of the participants’ commitment to improving the health of their rural patients. “Conditions” refers to the challenging social, physical and professional circumstances in which the physicians live and work, and “Corruption” signposts the broken promises given to the physicians and the unethical governance processes they have witnessed negatively impact on the commitment and conditions for the RR medical workforce and the health of local people.

## 4. Discussion

This study describes positive and negative experiences of physicians living and working in RR Indonesia. Positive experiences include appreciation from the RR communities and financial incentives earned. The negative experiences, more predominantly, included unfair treatment, late payments and ongoing manipulation, the lack of professional support and inadequate training and working facilities. Poor infrastructure, inadequate medical facilities and equipment, inadequate transport, lack of electricity, insufficient housing, and scarce clean water supply were other negative experiences described in this study. The data indicates a culture of corruption as an overarching theme, with evidence seen across all themes. Although the findings highlight some experiences that attracted physicians to RR Maluku and helped retain them, participants’ motivation to serve and stay in the RR workforce was negatively inhibited and eroded by these experiences.

Corruption in Indonesian law is expressed as unethical acts that harm the state’s finances or the state’s economy, and hinder national growth and development that demands high efficiency [32,33]. Included in these acts are embezzlement, fraudulent acts, extortion, abuse of power, gratification, bribery, and conflicts of interest in procurement [32,33]. In this study, corruption was strongly identified through fraudulent acts, abuse of authority, and conflicts of interest in procurement which combined with poor governance, living and working conditions to influence physicians’ motivation to remain in Indonesia’s RR districts.

### 4.1. Poor Governance

The findings of this study in terms of governance are consistent with other studies. The practice of poorly qualified leaders at the district level seems to be made possible by regional autonomy. This autonomy supports the notion of decentralised governance, authorising the regency and cities to regulate and manage their government affairs in the interests of their local community as per local statutory regulations [34]. However, the findings of this current study identify unethical practices, low accountability and a high lack of compliance to standards of consistent and fair treatment, disclosure and transparency. Similarly, in their study, Kristiansen and Santoso [24] found the local administration of health care services in four districts in Indonesia undergoing decentralisation (Bantul, Mataram, Kutai Kartanegara and Ngda) to have limited transparency and accountability. They reported that health centres are focused on creating profit. The increasing roles of private investors result in reduced concerns about preventive health care and the health status of poorer people [24]. Poor governance and corruption have been reported as significant challenges globally, especially in the low-and middle-income countries (LMICs), including Indonesia [35]. A 2020 scoping review of the influence of corruption and governance in the delivery of frontline health care services in the public sector identified a lack of institutional capacity and political commitment as a constraint to implementing regulation in the south and south-east Asian LMICs [35]. 

The Indonesian government has aimed to resolve workforce shortages through decentralisation, enabling regional autonomy [27]. Instead, more impoverished RR areas in the districts continue to suffer from shortages [1]. The findings reported here highlight how the district government is failing to address the medical workforce shortage with short-term solutions. On the one hand, there is poor oversight of health care provision of the district government despite the purpose of decentralisation [12,16]. On the other hand, the ongoing NS and WKDS programs indicate that the central government is reluctant to release the funds for the local government arrangement. Rather than bringing mainly urban-background physicians to the remotest regions with workforce outcomes that are not sustained, the central government could assign the role to the local government to bring local background medical graduates to the district. One strategy successful elsewhere is through education, building a “rural health pipeline” for sustainable recruitment and retention of rural physicians, supported by regional medical schools with a rural medical education program [36,37,38,39]. This strategy has successfully improved recruitment and retention of RR medical workforce globally. However, even this is unlikely to succeed without a commitment to good governance and transparency in recruitment and conditions. 

### 4.2. Working and Living in RR Areas

Neglect and abandonment were described as occurring in most working and living aspects, such as in service provision, purchasing and construction of infrastructures, facilities, equipment and supply. This undoubtedly impacts the physicians’ lives and ability to provide best-practice care and inevitably affects patient care and outcomes. Bruckner [40] describes corruption in the healthcare sector as a pandemic happening globally. He discusses how procurement of government goods/services in healthcare is greatly affected by corruption [40]. In the Indonesian context, this same form of corruption is evident in the number of government projects with delays that are not on target, not of the right quality, and not on budget [41]. Many buildings collapse after only reaching 30–40 percent of the expected life of a building due to non-compliance with or inadequate provisions in the technical specifications [41]. 

Participants in this study repeatedly identified that low-quality equipment and facilities, delays in providing goods, and budgeting politics relating to regional/district head elections hindered the delivery of quality health and medical services to the RR community. Budgets for public hospitals and primary health care in Indonesia come from the central and local governments [24]. However, varying conditions and quality of the facilities resulting in geographical disparities between Indonesian regions [12]. At the local government level, budgeting politics and the quality of budgeting are dependent on competing priorities. Most local government plans and budgeting do not prioritise quality health care and equity or recognise the social determinants of health [16]. 

Low commitment to and disregard for health workers’ rights were also evident in low and late payments and work insecurity. While physicians in rural Indonesia were fending for themselves in order to survive in a poor working environment, they believed their leaders did not mitigate the threats and dangers of the working environment. Although this situation remains undisclosed in official reports, this study reveals its negative impact on the motivation of previously devoted physicians to stay rurally. 

Physicians are becoming reluctant to work in remote areas because of the absence of reasonable living conditions. Furthermore, the under-functioning of the majority of health care facilities in such areas can erode their skills. Unless political action of the district government is executed, a poor RR working and living environment will maintain the reluctance of medical graduates to service RR areas, and the cycle of poor RR workforce will continue. A successful strategy in recruiting and retaining the medical workforce through collaboration with Regional Medical Schools to establish and maintain a rural pipeline [39] could improve local government investment in the healthcare sector. Medical Schools could support controls to ensure the government provides standard conditions for rural training and working and potentially help oversee their implementation.

### 4.3. Career and Professional Development

Poor quality of internship training due to inadequate facilities and supervision were identified in this study as detracting factors for post-interns to continue work rurally. Medical interns in another province of Indonesia also experienced this challenge. In rural and remote West Sumatra, medical interns experienced poor quality of communication in supervision and a lack of professional support and financial reward, which led to demotivation to continue practising rurally [42]. While the rural and remote internships in Indonesia may have been enabled access to medical services, it is not working to motivate physicians to stay to improve the medical workforce shortage in rural areas in the longer term. 

Generally, two alternative career pathways exist for a person employed as a government physician in Indonesia: following a postgraduate degree to become a specialist or promotion to an administrative position. Following the specialist path, physicians will not be able to remain in RR *Puskesmas*, as specialist training is only provided in cities with medical schools and advanced hospitals. Additionally, a specialist must be employed at least in a type-D hospital in the district’s centre [43]. Meanwhile, more senior administrative positions require physicians to be placed in the DHO in the center of the districts. Both pathways add to rural workforce turnover.

Additionally, nepotism and abuse of their power by those in leadership positions restrict development opportunities for physicians. Opportunities to enhance their professional knowledge and skills remain limited when the RR environment does not allow for professional development. The Rural Training Pathway (RTP), established in some countries with RR workforce shortages, such as Australia, supports RR physicians in ongoing and postgraduate training without leaving RR practice [44,45]. A Rural Generalist is a GP with a broad range of skills who specialises in rural practice [44,45]. However, despite its success in developing and sustaining RR medical workforce elsewhere, the Rural Generalist is not recognised in Indonesia. Providing and recognising such postgraduate training for RR physicians in Indonesian rural areas would be worthwhile to implement through collaboration with regional medical schools and the Indonesian Medical Council. 

We identified an absence of professional support for RR physicians and little commitment by senior managers to provide quality service in medical and health care. The RR medical workforce requires a support structure to function effectively [46,47]. Studies recommend support that includes mentoring in the workplace, adequate facilities, appropriate equipment for service and educational purposes, telecommunications sufficient to support access to urban specialist expertise, proper relief from on-call work and professional career development opportunities in RR areas [46,47]. As evidenced in this study, poor leadership has limited professional development opportunities for physicians working in Maluku RR districts. Political actions are required to make the recommended support a reality.

### 4.4. Motivation

The conditions of working and living in RR influenced physicians’ intention to serve the RR areas are identified through their statements of motivation. In this study, physicians who subsequently left RR practice mentioned the internal driver of idealism. They had initially intended to serve RR Maluku and wanted to see and make changes, but the circumstances did not enable it. Some felt they would not survive if they were subject to continued poor leadership and management, which necessitated seizing other job opportunities rather than continuing in the RR workforce. Musinguzi et al. [48] concluded from their study of health workers in Uganda that transformational leadership positively impacts job satisfaction and provides stimulating motivation in a resource-limited setting. Therefore, promoting transformational leadership at the district level in Indonesian RR is required to maintain the idealism of RR physicians and improve their retention.

As well as altruistic motivations shown by physicians in this study, the monetary gain was prominent as an additional consideration, particularly among younger physicians. However, as Handoyo et al. suggest, interventions based on incentives have worked well for recruitment but not for retention [49]. These approaches are mainly behaviourist and do not have long-lasting effects unless internalised to become intrinsically meaningful [49]. Ironically, some district governments in Maluku still advertise large incentives to attract physicians to their RR places without improving RR living and working environments. While this strategy may assist with the recruitment, retention of the RR physicians will remain challenging.

Some physicians who remained in Maluku RR work ultimately experienced the same workplace environment as those who left; however, they stayed for altruistic reasons [50]. Some of the physicians enjoyed the benefit of saving more money from the incentives, being recommended for a scholarship for postgraduate training by the district government, and hoping for career development in the district. While some hoped they could be the agent of change in the circumstances they encountered, others experienced no struggle because RR is their home, a place where they were born and raised; they completed a life cycle and derived comfort from their rural background [51]. This qualitative analysis confirms the findings of other studies that rural background plays a role in retaining physicians in remote places [52,53,54,55]. The evidence-based strategies described elsewhere regarding rural background should be implemented to recruit and retain physicians in RR Indonesia. 

We summarise recommendations and some potential means of implementation in response to the reported issues within the superordinate themes of this study. (Table 3). 

### 4.5. Limitations

This study explored in-depth the experience of physicians who have worked in RR areas with a larger sample size than many IPA studies. It offers rich and meaningful insights into participants’ experiences. Still, it could not distinguish the relative influence of the different aspects of governance, working and living conditions, career development and motivations for physicians’ decisions. The degree of influence is not solely contributed by the calculation of recurrent themes, as the degree of recurrence depends on the level of commenting and theming [25], and individual physicians will attribute different significance to individual factors. We also acknowledge that the authors’ familiarity with the topic may have resulted in some bias during the interpretation. However, the authors have been mindful of setting aside personal biases and consistently interpreting the participants’ perspectives to ensure credibility.

## 5. Conclusions

Multiple factors influenced physicians’ decisions to continue practising in RR areas of Indonesia and hindered the recruitment and retention of physicians in RR districts. Addressing the RR workforce shortage requires political action to reduce corruptive practices in the district governance. Focus on quality supply chain management, effective human resources, appropriate living conditions, and proactive management of career and education opportunities will contribute to a robust RR health service functioning with integrity. In doing so, influential leaders and supportive managers may be able to develop better living and working conditions conducive to physicians considering practising in RR communities. Additionally, a partnership with regional medical schools needs to be established to implement evidence-based strategies to improve workforce recruitment, development and retention.

## Figures and Tables

**Table 1:** List of interview questions.

Questions	Prompts
Can you tell me about your experiences living and working as a physician in Maluku Province’s rural and remote areas?	What is the interesting and memorable experience during your work as a RR physician?
	What are the good things about working and living in RR areas? *
	What are the bad things about working and living in RR areas? *
Can you tell me what factors contributed to your choice of work and live as a physician in rural Maluku?	What factors made you decide to stay or leave the rural and remote practice in Maluku?
	How did you arrive at your decision? *
Can you tell me about your motivation to live and work rurally as a physician in Maluku?	What are the most significant reasons you want to serve in a RR practice?

* developed based on the example from Smith, 2009 [25].

**Table 2:** Characteristics of the respondents.

Alphanumeric Code	Employment Status	Workplaces	Work Location	Rural Background
PI1	Permanent	District hospital and community health centre	Rural	No
PI2	Temporary	District hospital and community health centre	Rural	No
PI3	Permanent	District hospital and community health centre	Rural	No
PI4	Permanent	District hospital and community health centre	Rural	No
PI5	Temporary	District hospital and community health centre	Rural	No
PI6	Temporary	District hospital and community health centre	Rural	No
PI7	Temporary	District hospital and community health centre	Rural	No
JD1	Temporary	Community health centre	Remote	No
JD2	Permanent	Community health centre	Remote	No
JD3	Permanent	Community health centre	Remote	No
JD4	Temporary	Community health centre	Remote	No
JD5	Permanent	Sub-district hospital	Remote	No
JD6	Temporary	Community health centre	Remote	No
JD7	Permanent	District hospital	Rural	Yes
JD8	Permanent	Community health centre	Remote	No
JD9	Temporary	Community health centre	Remote	No
SD1	Permanent	District hospital	Rural	No
SD2	Temporary	Community health centre	Remote	No
SD3	Permanent	District hospital	Rural	No
SD4	Permanent	Community health centre	Rural	Yes
SD5	Permanent	Community health centre	Remote	No
SD6	Permanent	District hospital	Rural	No
SD7	Permanent	Community health centre	Rural	No
SD8	Permanent	Sub-district hospital	Remote	Yes
S1	Permanent	District hospital	Rural	No
S2	Permanent	District hospital	Rural	No

**Table 3:** Challenges and recommendations.

Reported Issue	Recommendation	Potential Means for Implementation
*Poor governance*		
Favouritism, complicated bureaucracy, fraud and bogus offers, negligence, money-orientation	Establish oversight by central (or provincial) government Standardise pay and conditions Ensure contracts with appropriate conditions specified and registered with an agency charged with oversight Undertake exit surveys and interviews with a requirement that all services report findings Ensure adequate personal safety and clinical governance	Appoint a rural health commissioner or ombudsman for complaints and proactive oversight of adherence to mandatory standards
Prioritising temporary physicians (Nusantara Sehat, WKDS)	Require districts to provide their own health human resources through the “Rural Health Pipeline” for sustainable recruitment and retention of rural physicians	Request or require medical schools to assist with outreach and selection of future physicians from rural districts.
*Career and professional development*		
Poor quality of internship training due to inadequate facility and equipment and limited supervision	Implement audit of the hospitals and primary care centres involved in internship to comply with the mandatory standard facility and equipment, and supervision for the internship program	Develop a strategy for audit and upgrade as necessary of all RR clinics and hospitals involved in the internship program, including remote connectivity for education and clinical support
Inadequate opportunities for professional development	Payment of additional funding to remote health practitioners for professional development with two weeks of paid professional development leave. Create opportunities for ongoing training of physicians seeking to become rural generalists and opportunities for relevant specialist	Establish a working party to examine options for ongoing professional development for remote physicians. Support from local universities, professional bodies and the Indonesian Medical Council to develop appropriate models for training applicable for remote physicians (e.g., Rural Training Pathways, rural generalist)
*Living and working conditions*		
Inadequate equipment for medical work	Implement audits of hospitals and review of adherence to accreditation standards	Develop a strategy for audit and upgrade as necessary of all RR clinics and hospitals
Poor living conditions (electricity, housing, water supply, road and transportation)	Standards established for conditions for housing Additional investment by governments in appropriate and secure housing for the health workers in remote communities	Develop a strategy for audit and upgrade as necessary of all health worker accommodation in remote settings Regular assessment and reporting of health practitioner housing stock in remote settings with professional oversight to ensure standards are being met. Medical schools can undertake the controlling mechanism to ensure the government provides standard conditions for rural training and working/living.

## Data Availability

Not applicable.
